# Side Chain Geometry Determines the Fibrillation Propensity of a Minimal Two-Beads-per-Residue Peptide Model

**DOI:** 10.1021/acs.jpcb.2c03502

**Published:** 2022-08-02

**Authors:** Beata Szała-Mendyk, Andrzej Molski

**Affiliations:** Faculty of Chemistry, Adam Mickiewicz University in Poznań, Uniwersytetu Poznańskiego 8, 61-614 Poznań, Poland

## Abstract

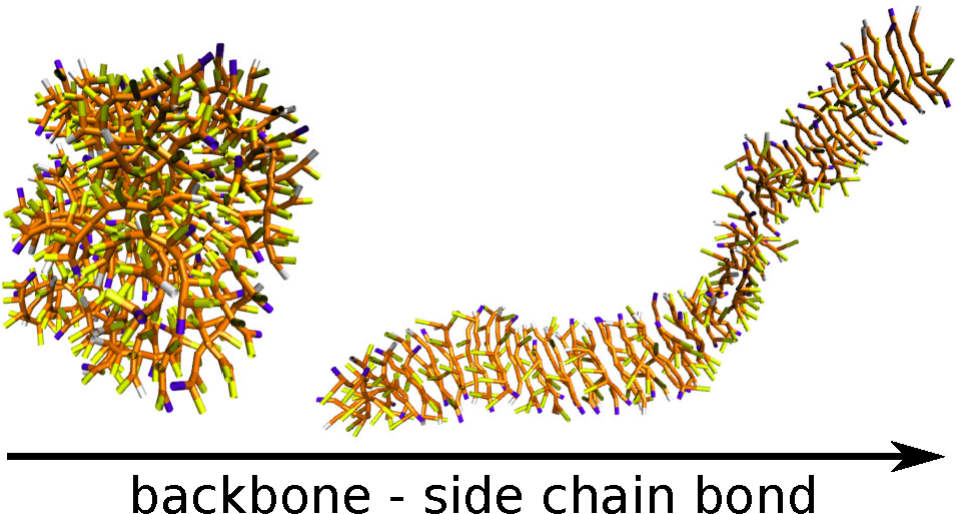

The molecular mechanism of fibrillation is an important issue for understanding peptide aggregation. In our previous work, we demonstrated that the interchain attraction and intrachain bending stiffness control the aggregation kinetics and transient aggregate morphologies of a one-bead-per-residue implicit solvent peptide model. However, that model did not lead to fibrillation. In this work, we study the molecular origin of fibril formation using a two-beads-per-residue model, where one bead represents the backbone residue atoms and the other the side chain atoms. We show that the side chain geometry determines the fibrillation propensity that is further modulated by the modified terminal beads. This allows us to bring out the effects of side chain geometry and terminal capping on the fibrillation propensity. Our model does not assume a secondary structure and is, perhaps, the simplest bead-based chain model leading to fibrillation.

## 1 Introduction

The molecular mechanism of fibrillation is an important issue for understanding peptide and protein self-assembly. Aggregation of proteins and peptides into fibrillar structures is connected with neurodegenerative diseases and systemic pathologies,^1^ and with potential application in materials science.^2^ It is difficult to follow the molecular details of this process with current experimental methods. Computer simulations are often applied to study protein aggregation at different resolution levels, from coarse-grained to atomistic.^3^

Coarse-grained models for protein aggregation are commonly used to expand the simulation time and increase the system size.^4^ One type of coarse-grained model presents peptides as chains of superatoms where each peptide residue is mapped to one superatom.^5−7^ Interestingly, such simple models can reproduce a variety of equilibrium structures observed in experiment. For instance, in a series of papers, Janke and colleagues studied the thermodynamics of peptide aggregation using a homopolymer model.^5,8,9^ Ranganathan et al. connected the interaction strength, bending stiffness, and polymer chain length with various signatures of protein aggregation and amyloid formation.^6^ Coarse-grained models with two or three beads per residue have been successfully applied to study proteins and peptides behaviors such as folding, aggregation, and fibrillation.^3^ Sequence-specific models, such as UNRES, give insight into the aggregation of selected peptides, depending on their amino acid content.^10,11^ On the other hand, nonspecific models allow investigating general properties of biological aggregation. For example, the models proposed by Caflisch and co-workers^12^ and by Bellesia and Shea^13^ show how the β-sheet propensity may influence the structure of peptide aggregates.

In our previous work, we demonstrated that the interchain attraction and intrachain bending stiffness control the aggregation kinetics and transient aggregate morphologies of a one-bead-per-residue implicit solvent peptide model.^7,14^ However, that model did not lead to fibrillation. In this work, we study the molecular origin of fibril formation using a new two-beads-per-residue model, where one bead represents the backbone residue atoms and the other the side chain atoms. Our model may represent intrinsically disordered homopetides: polyalanine, polyasparagine, or polyglutamine. We address a major question: What are the minimal requirements for a bead-based model of a peptide chain to give rise to fibrillation? Specifically, we study the effects of side chains and terminal beads. Our preliminary simulations revealed, for instance, that the breaking of residue symmetry by the presence of side chains can lead to the formation of short fibrils. When the end-beads are different than those along the chain, one can see the formation of longer and more stable fibrils.

## 2 Methods

**Figure 1:**
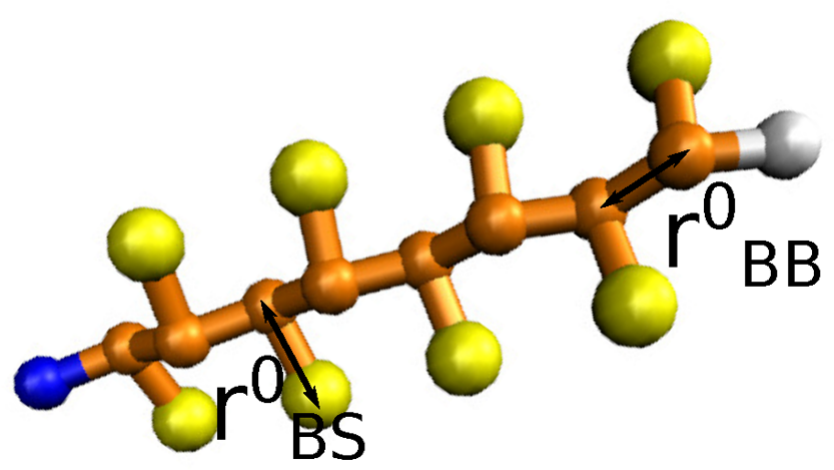
Peptide model with 8 residues, R8, with two superatoms per residue and modified terminal superatoms. The yellow beads represent side chain superatoms, S; the orange beads represent backbone superatoms, B, and the blue and white beads correspond to the terminal caps, T.

The present model assumes three types of superatoms: backbone (B), side chain (S), and terminal cap (T) superatoms. See Figure 1.

The bonded interactions between adjacent superatoms are harmonic:
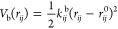
1where the indexes *i*, *j* represent two adjacent bondend superatoms, and *k*_*ij*_^b^ and *r*_*ij*_^0^ are the force constants and equilibrium bond lengths, respectively, that depend on the types of connected superatoms. For the backbone bonds and terminal cap bonds, the force constants are fixed at *k*_BB_^b^ = *k*_BT_^b^ = 1250 kJ mol^–1^ nm^–2^, and the bond lengths are fixed at *r*_BB_^0^ = *r*_BT_^0^ = 0.35 nm. The force constant for the backbone–side chain bond is also fixed at *k*_BS_^b^ = 5000 kJ mol^–1^ nm^–2^, whereas the equilibrium length of this bond, *r*_BS_^0^, is a scanned parameter and changes in the range 0.40–0.70 nm. The fixed bonded parameters, *r*_BB_^0^, *r*_BT_^0^, *k*_BB_^b^, *k*_BT_^b^, and *k*_BS_^b^, are set according to the MARTINI model, an often used coarse-grained peptide model with similar molecular resolution.^15^ The parameter range for *r*_BS_^0^ is based on our preliminary simulations.

The angle potentials between adjacent bonds are modeled as
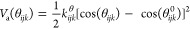
2where the indexes *i*, *j*, and *k* represent three consecutive superatoms, and θ_*ijk*_^0^ is the equilibrium bond angle fixed at θ_BBB_^0^ = 150° and θ_BBS_^0^ = 105° for the angles between BBB and BBS superatoms, respectively. The angle force constant is fixed at *k*_*ijk*_^θ^ = 1000 kJ/mol for both backbone (BBB) and backbone–side chain superatom (BBS) angles. The angle parameters are based on our previous work,^7^ where we showed that ordered aggregates are formed by stiff chains, *k*_*ijk*_^θ^ ≥ 800 kJ/mol. The equilibrium backbone angle was modified to take into account the presence of side chain superatoms.

The nonbonded interactions are defined by Lennard-Jones potential
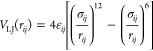
3and the modified Lennard-Jones potential that mimics the repulsive interactions between identical termini
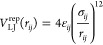
4where *r*_*ij*_ is the distance between two nonbonded superatoms, ε_*ij*_ is the depth of potential minimum, and σ_*ij*_ is the Lennard-Jones radius. The LJ parameters σ_*ij*_ and ε_*ij*_ vary depending on the interacting superatom pairs, see Table 1.

**Table 1:** Lennard-Jones Parameters for Different Pairs of Superatoms^a^

	side chains	backbone	N-termini	C-termini
side chains	0.37 ≤ σ_SS_ ≤ 0.45	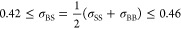	σ_ST_ = 0.40	σ_ST_ = 0.40
	ε_SS_ = 1.0	ε_BS_ = 2.0	ε_ST_ = 0.5	ε_ST_ = 0.5
backbone		σ_BB_ = 0.47	σ_BT_ = 0.40	σ_BT_ = 0.40
		ε_BB_ = 4.5	ε_BT_ = 0.5	ε_BT_ = 0.5
N-termini			σ_NN_^rep^ = 0.40	σ_NN_ = 0.40
			ε_NN_^rep^ = 4.0	ε_NC_ = 4.0
C-termini				σ_CC_^rep^ = 0.40
				ε_CC_^rep^ = 4.0

a The repulsive LJ interactions are marked by the superscript rep. The LJ radii σ_*ij*_ are expressed in nm, and the potential depths ε_*ij*_ are expressed in kJ/mol.

The values of the LJ interaction strength are chosen following Dobson et al.^16^ who suggested that the formation of β-rich fibrils is facilitated by the backbone–backbone interactions.^17^ On the basis of this observation, our model assumes that the backbone–backbone interactions are stronger then the backbone–side chain and side chain–side chain interactions, see Table 1. The charged termini behavior is accound for by the high interaction strength and the only repulsive potential for identical ends. The LJ radius for the backbone superatoms is taken from the MARTINI force field, whereas the range of the side chain LJ radius is based on our preliminary simulations. The LJ interactions are excluded only for pairs of superatoms connected via the harmonic bond potential.

The GROMACS 2020.5 package^18^ was used for all simulations. The dynamics was propagated with a leapfrog stochastic dynamics integrator which also serves as a thermostat at 303 K, and with periodic boundary conditions in all directions. The integration time step was 15 fs. All simulations were carried with implicit solvent, which is defined by the friction coefficient used with the stochastic dynamics integrator. To mimic the friction effect of solvent, an inverse friction coefficient of 0.17 ps was applied. Our simulations concern the initial, nonequilibrium phase of peptide aggregation. The simulations were carried out for 35 systems that differ in two model parameters: the side chain LJ radius σ_SS_ = 0.37–0.45 nm and the equilibrium length of the bond of the side chain–backbone superatoms, *r*_BS_^0^ = 0.40–0.70 nm. A single simulation is 12 μs long. For each system, five independent repeats were performed, giving 60 μs per system. Each repeat started with 72 monomers randomly placed in cubic box with the edge length *L* = 35 nm. When reporting on repeated simulations, we show the standard deviation of the mean for the kinetic curves.

Some additional simulations were performed for selected systems starting from initial 72-mer aggregates. The spatial arrangements of these initial aggregates were the spatial arrangements of final clusters in previous simulations. After an adjustment of the model parameters to new values, the clusters were watched for stability for 6 μs.

The definition of a cluster is based on a cutoff distance: A peptide belongs to a cluster if the distance between an atom of this peptide and an atom of a different peptide in the cluster is equal or less than 5.5 Å. This cutoff is the location of the first maximum on the distribution of atom distances in clusters, see Supporting Information Figure S1.

Structural features of aggregates were described by two descriptors: the end-to-end correlation parameter *C*_**n**_, and backbone–backbone correlation parameter, *C*_BB_. The end-to-end correlation parameter, *C*_**n**_, was introduced in the polymer physics literature to characterize the aggregation transition of polymer systems.^5^ It is defined as
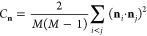
5where the unit vector **n**_*i*_ is the normalized end-to-end vector of the backbone atoms of peptide *i* and *M* is the cluster mass expressed as the number of peptides forming an aggregate. The parameter *C*_**n**_ describes the order of polymer chains in an aggregate and takes a value of 1 for the parallel alignment of chains and a value around 0.3 for the random orientation. *C*_**n**_ corresponds to the directional order of peptide chains, whereas the positional order is measured by the backbone–backbone correlation parameter, *C*_BB_, defined as
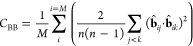
6where *n* is number of neighbors located within 0.8 ≤ *r* ≤ 2.4 nm from peptide *i*. This distance range has been chosen on the basis of the intermolecular backbone distance distribution, see explanation in Supporting Information Section S1.2.  and  are normalized vectors connecting backbone mass centers between peptide *i* and its neighbors *j* and *k*, respectively.
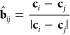
7Here, **c**_*i*_ and **c**_*j*_ are the backbone mass centers of peptide *i* and *j*.

Aggregation kinetics were investigated by following the number of free monomers, *n*_1_, dimers, *n*_2_, trimers, *n*_3_, and the total number of clusters including monomers, *n*_c_ = ∑_*i* = 1_^*N*^*n*_*i*_. We found that the Smoluchowski-type kinetic model,^19^ where all clusters aggregate irreversibly with equal rate constants, is not appropriate for our simulations. Accordingly, we have modified that model by allowing for fragmentation with a constant rate and assuming that monomers aggregate and dissociate faster than the other clusters.

In stochastic models of aggregation kinetics,^20,21^ the rates of the aggregation events, *C*_*i*_ + *C*_*j*_ → *C*_*i*+*j*_, and fragmentation events, *C*_*i*+*j*_ → *C*_*i*_ + *C*_*j*_, are proportional to the number of aggregating pairs and the number of fragmenting clusters, respectively. The proportionality constants are the aggregation and fragmentation rate constants, *k*_*i*,*j*_^+^ and *k*_*i*,*j*_^–^, respectively. Those rate constants are size-dependent. We adopt a convention that the aggregation rate between different-size clusters, *i* ≠ *j*, is *k*_*i*,*j*_^+^*c*_*i*_*c*_*j*_, and that for equal-size clusters, *i* = *j*, is . The factor 1/2 in the aggregation rate for equal-size clusters is motivated by the fact that the number of (*i*, *i*) pairs is ≈*c*_*i*_^2^/2. A different convention, where the factor 1/2 is combined with *k*_*i*,*i*_^+^, i.e., , is also used.^22^

We used the following modified Smoluchowski rate equations that account for the finite system size and aggregation reversibility:
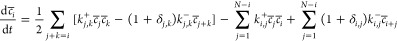
8where  is the trajectory-averaged concentration of clusters made up of *i* monomers, and δ_*i*,*j*_ is the Kronecker delta. The factor 1/2 prevents double-counting in the sum *∑*_*j*+*k*=*i*_. The factors (1 + δ_*i*,*j*_) account for the stoichiometric factors when two identical clusters are involved, 2*C*_*i*_ → *C*_2*i*_ and *C*_2*i*_ → 2*C*_*i*_.

A stochastic kinetic model is fully specified when the size-dependent rate constants, *k*_*i*,*j*_^+^ and *k*_*i*,*j*_^–^, are given. Our kinetic model involves three fit parameters: the aggregation rate constant, *k*^+^, for all cluster pairs except for monomer association, the fragmentation rate constant, *k*^–^, for all clusters except for dissociation of monomers, and the ratio *q* of the monomer association rate constant and *k*^+^, that is equal to the ratio of the monomer dissociation rate constant and *k*^–^. In terms of size-dependent rate constants, our three-parameter model is defined as *k*_*i*,*j*_^+^ = *k*^+^ and *k*_*j*,*i*_^–^ = *k*^–^ when *i*, *j* ≠ 1, and *k*_*i*,*j*_^+^ = *qk*^+^ and *k*_*j*,*i*_^–^ = *qk*^–^ when *i* or *j* = 1. Note that this simple model preserves detailed balance.

Equation 8 was solved numerically for the numbers of clusters . The total number of cluster was calculated as . The number of monomers, dimers, and trimers and the total number of clusters were globally fit to the averaged simulation curves by minimizing the sum of the squared differences between the simulation data and model.

When the compartment where aggregation occurs is small, e.g., in biological cells or simulation boxes, the numbers of aggregating species may be so low that number fluctuations become significant. Therefore, the application of rate equations to the cases involving small numbers of oligomers is only an approximation, and it is important to validate the rate models by comparing them to their stochastic counterparts. For each set of the fit parameters, *k*^+^, *k*^–^, and *q*, we generated 1000 stochastic repetitions of the aggregation-fragmentation kinetics using the Gillespie algorithm.^23,24^ We compared the averaged stochastic kinetics, , , , and , to the fitted kinetics *n*_1,fit_, *n*_2,fit_, *n*_3,fit_, and *n*_c, fit_, and found that, in the present case of *N* = 72 monomers, the averaged stochastic kinetics are well-represented by the modified Smoluchowski equations. For instance, the corresponding kinetic curves overlap within the resolution as shown in Figure 5 and Supporting Information Figure S6 (data not shown).

## 3 Results and Discussion

**Figure 2:**
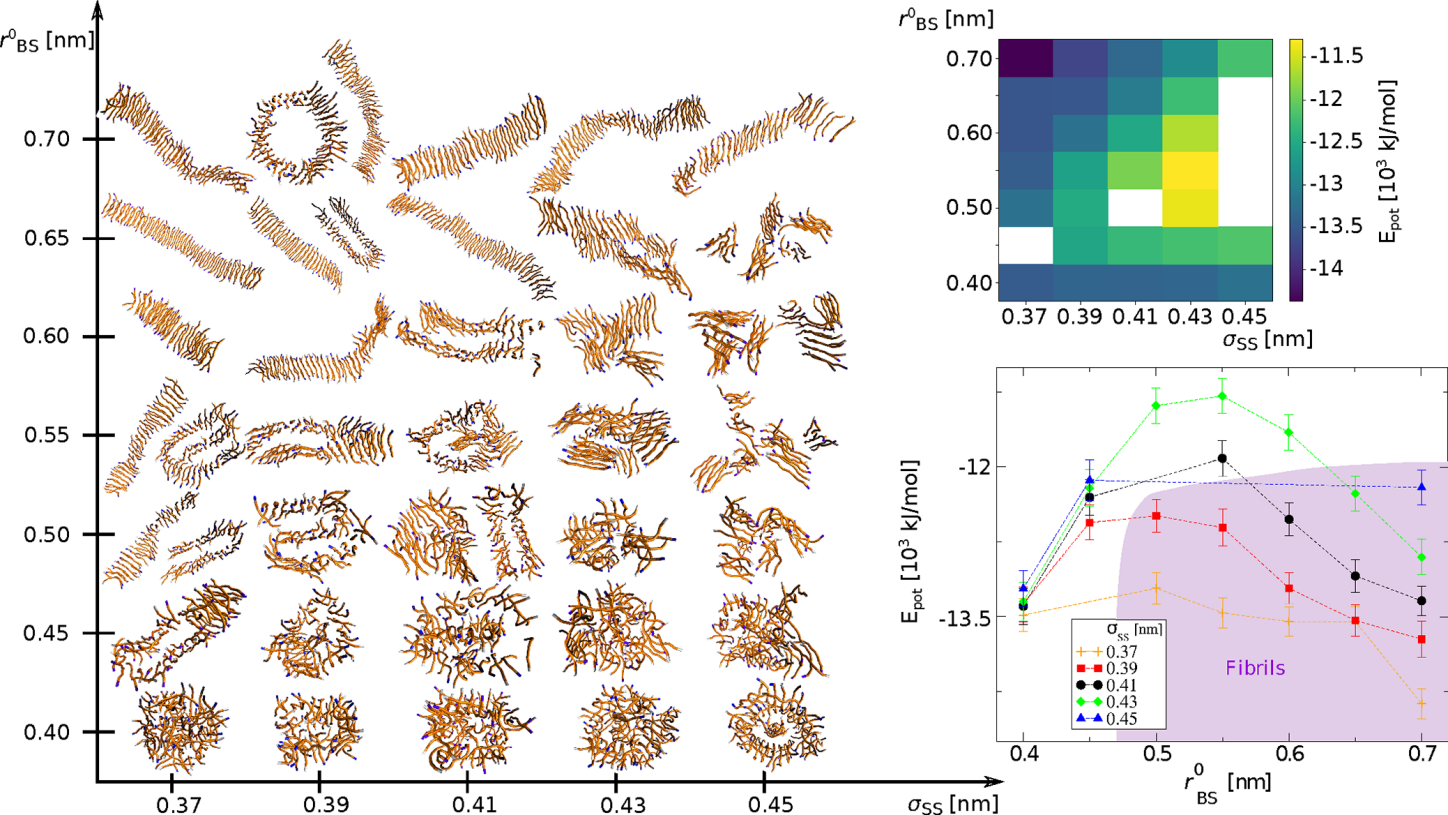
(Left panel) Structural diversity of aggregates as a function of two model parameters determining the side chain geometry: the length of the bond between backbone and side chain superatoms, *r*_BS_^0^, and the Lennard-Jones radius, σ_SS_, of side chain superatoms. Note that σ_BS_ changes according to Table 1. The snapchots present the maximal cluster, *M* = 72, for all except 5 systems where the maximal cluster did not appear: σ_SS_ = 0.37 and *r*_BS_^0^ = 0.45 nm, *M* = 58; σ_SS_ = 0.41 and *r*_BS_^0^ = 0.50 nm, *M* = 57; σ_SS_ = 0.45 and *r*_BS_^0^ = 0.50 nm, *M* = 56; σ_SS_ = 0.45 and *r*_BS_^0^ = 0.55 nm, *M* = 52; σ_SS_ = 0.45 and *r*_BS_^0^ = 0.60 nm, *M* = 59; σ_SS_ = 0.45 and *r*_BS_^0^ = 0.65 nm, *M* = 55. (Right panels) Average value of potential energy for the maximal aggregates, *M* = 72, for different model parameters. The maximal aggregate, *M* = 72, has not been formed for a few systems, which is seen as white squares in the top right plot and as “missing points” in the bottom panel.

The structural diagram presented in Figure 2 shows how the aggregate morphology is determined by the side chain geometry. Peptides with short backbone–side chain bonds tend to form mostly amorphic, spherical aggregates, as seen at the bottom of the diagram. Such aggregates may show some local order, especially when the side chain size increases. When the length of the backbone–side chain bond increases, the peptides take parallel orientation. A common unit observed for most ordered aggregates is an antiparallel peptide pair. This directional order is only short-distance. The lack of long-term order leads to the formation of the glass-like aggregates, especially for peptides with larger side chains, σ_SS_ = 0.41, 0.43, and 0.45 nm. This novel phase, called an amyloid-glass, has been recently postulated by Mioduszewski and Cieplak in their computational study of proteinaceous liquid droplets.^25^ In an amyloid-glass, peptide chains take parallel or antiparallel orientation relative to their neighbors, which leads to the formation of small, amyloid-like, arrangements. However, these arrangements are randomly placed within aggregate; thus, the overall morphology is quite amorphous. Mioduszewski and Cieplak found the temperature-dependent transition between liquid and amyloid-glass phases, where the latter appears at low temperature for longer peptide chains. Our results complement that finding and introduce the side chain geometry as an additional factor causing amyloid-glass formation. The LJ radius seems to be especially important, as for the smallest side chain radii glass-like aggregates are not formed at all.

For the smaller side chain sizes, σ_SS_ = 0.37, 0.39 nm, with the medium length of the B–S bonds, *r*_BS_^0^ = 0.50 nm, different fibrilar structures are observed: one-ribbon fibrils, bend fibrils stabilized by side chain interactions, or even elongated cylinders filled by side chains. For longer B–S bonds, the fibril-like structures are observed for all side chain sizes. Depending on the side chain size and the bond length, the fibrils may consist of one or more ribbons and have different flexibility, as is seen at the top of the phase diagram, Figure 2.

Interestingly, for the system with σ_SS_ = 0.39 and *r*_BS_^0^ = 0.70 nm, an annular structure was observed in one simulation repeat. This annular structure resembles amyloid ion channels that were experimentally observed for amyloid-β peptides,^26^ α-synuclein,^27^ and polyglutamine.^28^ There are a few models of this channel structure.^26^ A common feature is the presence of a U-shaped β-strand–turn−β-strand motif. Neighboring U-shaped peptides interact via hydrogen bonds to form two-layer β-sheet. A recent experimental study provides a more detailed insight into the internal structure of β-sheet pore-forming oligomers for amyloid-β(1–42) peptide.^29^ Ciudad and co-workers found that there are two cylindrical oligomers: tetramers and octamers that slightly differ in an internal peptide organization. In our simulations, octapeptides are too short to take a U-shaped structure; instead, they form the antiparallel pairs as the basic motif. These pairs interact with the neighbors to form also a two-layer cylinder. Though an annular structure was obtained only for system with *r*_BS_^0^ = 0.70 nm and σ_SS_ = 0.39 nm, we examined if it would be stable also for other side chain sizes. We conducted an additional simulation set for the constant bond length, *r*_BS_^0^ = 0.70 nm, and different side chain sizes, σ_SS_ = 0.37, 0.39, 0.41, 0.43, and 0.45 nm, during 6 μs. All simulations started from the annular structure obtained earlier for *r*_BS_^0^ = 0.70 nm and σ_SS_ = 0.39 nm. On the basis of this simulation set, we found that the annular structure is stable for the small side chains, σ_SS_ = 0.37, 0.39, and 0.41 nm, for the whole simulation time. In these cases, the potential energy of the annular structure is slightly lower than that for the corresponding fibrils. For the larger side chains, the annular structure collapses to a fibril with two protofilaments whose potential energy is quite similar to that for single-ribbon fibrils.

The significance of this observation is connected with the cytotoxicity shown by amyloid ion pores.^30^ The morphological features of annular protofibrils, as well as their membrane-affinity, make them similar to the β-sheet pore-forming protein toxins which increase the membrane permeabilization leading to changes in cell activity or even to cell death.^27,31^ The annular protofibrils have been pointed out as not necessary for fibril formation, and they can coexist with fibrils in molecular-crowded environments.^27^ Interestingly, our results indicate that the competition between fibrils and annular structures is dependent on the LJ radii of side chains, and the stability of annular structure decreases for larger side chain LJ radii.

For simulation runs that lead to a single (= maximal, *M* = 72) cluster, we measured the average potential energy of the final cluster; see the right panels in Figure 2. The transition from amorphous aggregates formed for the lowest *r*_BS_^0^ to glass-like structures is associated with an increase in the potential energy. The potential energy is maximal for amyloid-glass structures, σ_SS_ = 0.41 with *r*_BS_^0^ = 0.50–0.55 nm, and σ_SS_ = 0.43 with *r*_BS_^0^ = 0.50–0.60 nm, which may suggest a metastable nature of these structures. The potential energy decreases for fibrillar structures. Interestingly, not all fibrilar aggregates assume the potential energy that is lower than that for the initial amorphous aggregates, especially the fibrils for larger side chains (σ_SS_ = 0.43, 0.45) have higher energy. The energy plot does not include all systems, as not in all cases a maximal cluster was formed, even when we extend simulation time to 18 μs.

To check if the amyloid-glass structures represent a thermodynamic trap, we performed an additional simulation set for the systems with σ_SS_ = 0.43 and whole range of BB–SC bond lengths, 0.40 ≤ *r*_BS_^0^ ≤ 0.70 nm. In this simulation set, the initial arrangement was taken as the amyloid-glass structure obtained previously as a final structure for a system with σ_SS_ = 0.43 nm and *r*_BS_^0^ = 0.55 nm. For each system, the initial glass-like assembly reorganizes into the structure similar to the final aggregate obtained by direct aggregation from randomly placed monomers. The amyloid-glass structure was stable during the whole simulation time only for systems with medium side chain–backbone bonds, 0.50 ≤ *r*_BS_^0^ ≤ 0.60 nm. For longer bonds, amyloid-glass undergoes a transition to double-ribbon fibrils for *r*_BS_^0^ = 0.65 nm or to an annular structure for *r*_BS_^0^ = 0.70 nm. On the other hand, for shorter bonds, *r*_BS_^0^ = 0.40 and 0.45 nm, the amyloid ribbons lose their ordered structure, and more irregular aggregates are obtained at the end of simulation.

To investigate the difference in the formation of amorphous and fibrillar aggregates, we use two structural descriptors which measure the positional and directional order of peptide chains in an aggregate. The directional order is measured on the basis of the end-to-end vectors defined for each peptide in the cluster, eq 5. The second parameter refers to the local positional order and measures the correlation of the vectors defined between the backbone mass centers of two neighbor peptides, eq 6.

**Figure 3:**
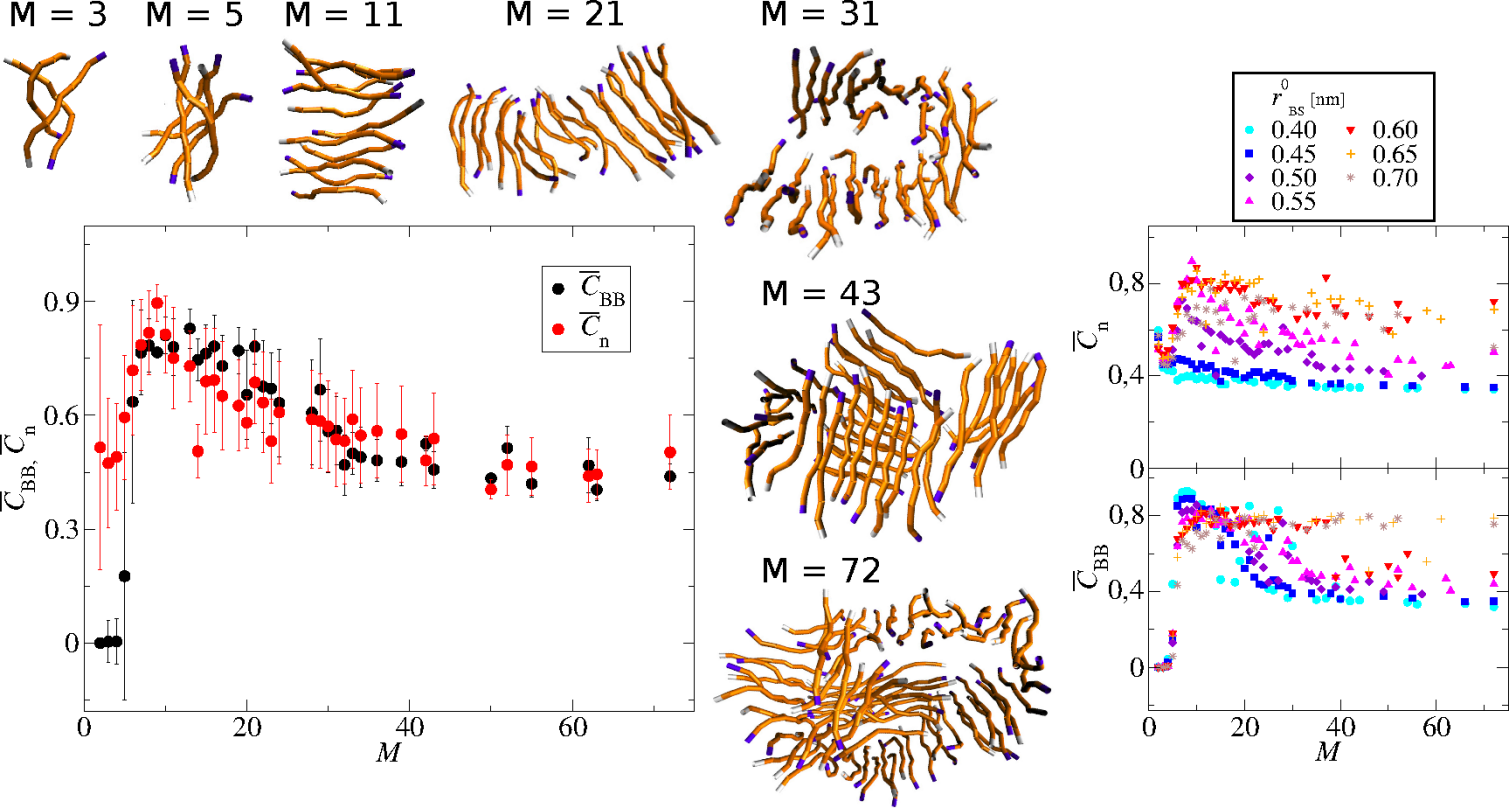
(Left panel) Average end-to-end correlation parameter, , and backbone correlation parameter, , as a function of the aggregate size, *M*, for side chain superatom LJ radii σ_SS_ = 0.41 nm and length of the backbone–side chain superatom bond, *r*_BS_^0^ = 0.550 nm, with example figures. (Right panel) Average end-to-end correlation parameter, , upper plot, and backbone correlation parameter, , bottom plot, as a function of the aggregate size, *M*, for side chain superatom LJ radii σ_SS_ = 0.41 nm and different lengths of the backbone–side chain superatoms bond as indicated. For better visualization, error bars are not shown; for the standard deviations, see Supporting Information Figure S5.

The structural analysis for the glass-like system, σ_SS_ = 0.41, *r*_BS_^0^ = 0.550 nm, is presented in the left panel of Figure 3. This analysis shows that the initial oligomers are the most ordered. These oligomers, with sizes in the range 5–20 peptides, have relatively large positional and directional order, which is seen as a maximum in this plot. Further coalescence of oligomers leads to the formation of glass-like aggregates with low order parameters, the positional and directional. We do not observe a rearrangement of these glass structures into fibrils during the simulation time.

The right panel of Figure 3 shows the average end-to-end correlation parameter, , and the backbone correlation parameter, , as a function of the aggregate size, *M*, for the same side chain size, σ_SS_ = 0.41, and different backbone–side chain bonds, 0.40 ≤ *r*_BS_^0^ ≤ 0.70 nm. For the short backbone–side chain bonds, the directional order is small for all aggregates, from oligomers to the final cluster. However, the initial oligomers, with mass up to 15 peptides, have a relatively high positional order. See also Supporting Information Figure S3 for an example analysis of an amorphous system with the corresponding fibrilar oligomers. These elongated structures are not stable for larger aggregates and undergo a transition into cylindrical or spherical structures. Further growth leads to the formation of amorphous aggregates with low positional and directional order.

**Figure 4:**
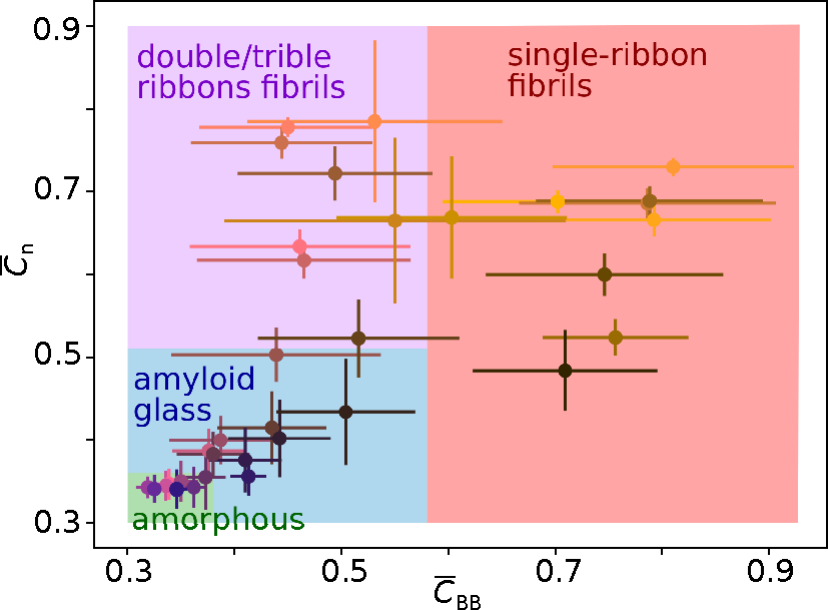
Average end-to-end correlation parameter, , and backbone correlation parameter, , calculated for the largest final aggregates. Color areas indicate type of aggregate structure: amorphous (green), amyloid-glass (blue), double/triple-ribbons fibrils or annular structures (purple), and one-ribbon fibrils (red).

On the other hand, peptides with long backbone–side chain bonds form directly ordered oligomers. The oligomer growth leads to fibrils without any structural reorganization; thus, the structural parameters reach the largest value for oligomers with size around *M* ≈ 10 and do not change much for larger aggregates. See Supporting Information Figure S4. A decrease of the end-to-end correlation parameter for some large fibrils is caused by the fibril twist. On the other hand, *C*_BB_ is lower for the fibrils with multiple ribbons than for single-ribbon fibrils. None of these parameters alone is sufficient to distinguish fibrillar from amorphous and glass-like aggregates. However, when we plot both parameters, we obtain a phase diagram with four groups of points, corresponding to four types of structures: amorphous aggregates, amyloid-glass, multiple-ribbon fibrils, and single-ribbon fibrils. See Figure 4. This analysis shows that the local positional order has higher values for single-ribbon fibrils than for any other structures. On the other hand, multiple-ribbons fibrils may have a higher directional order seen as the high values of the  parameter. Generally, the amyloid-glasses have a higher positional and directional order than amorphous aggregates, but an accurate borderline is difficult to define. This suggests that the transition between amorphous and glass-like aggregates is gradual rather than sharp. The amyloid-glasses within this blurred transition region may share many features of amorphous aggregates, making them difficult to distinguish in experiments. Elongation of the ordered fragments within amyloid-glass aggregates is paralleled with rising values of both order parameters,  and *C*_BB_, approaching glass systems to multiple-ribbon fibrils.

**Figure 5:**
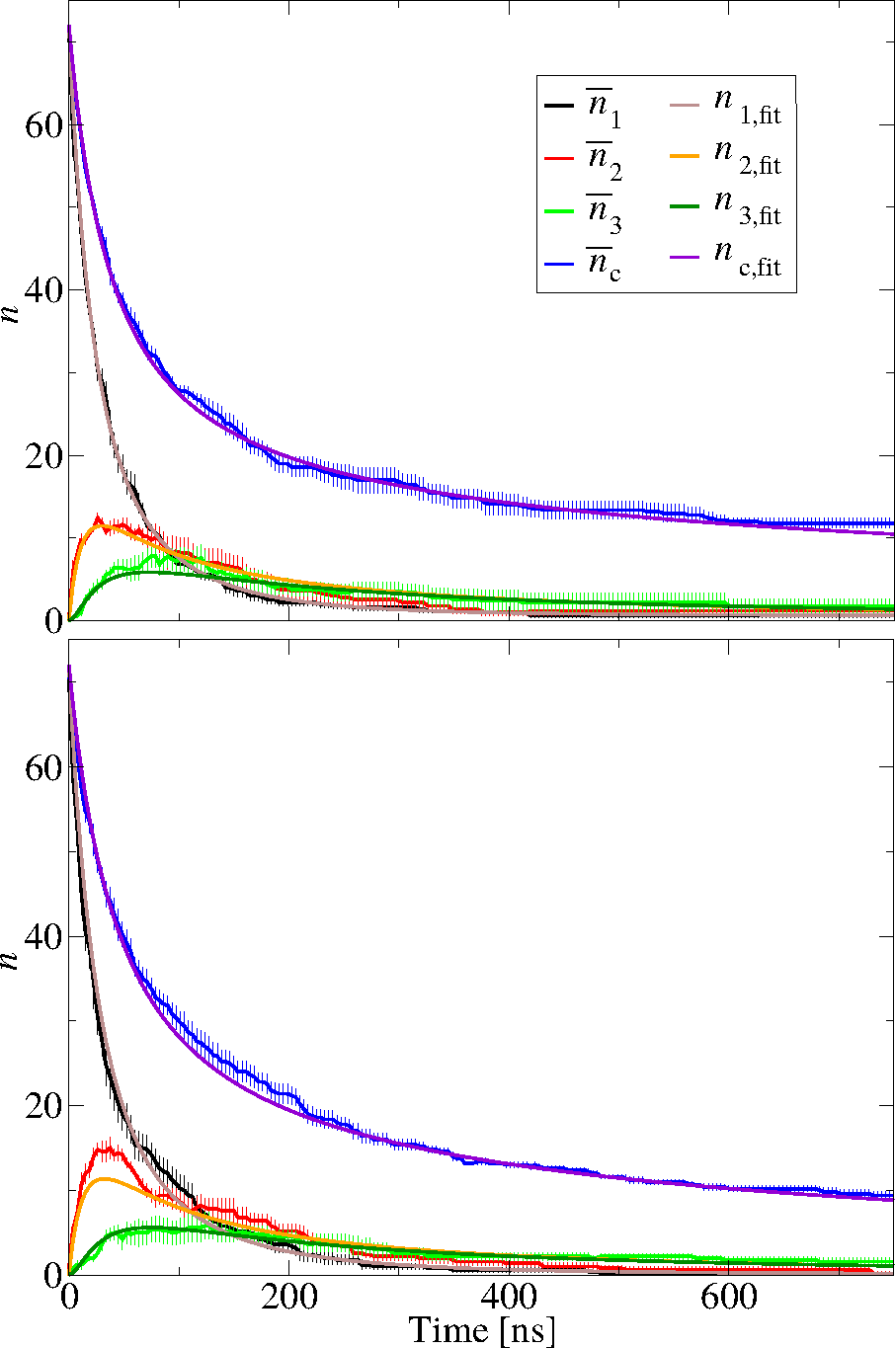
Kinetic plots for amorphous (σ_SS_ = 0.37, *r*_BS_^0^ = 0.40 nm, top panel) and fibrillar (σ_SS_ = 0.45, *r*_BS_^0^ = 0.70 nm, bottom panel) systems. Data from the MD simulations are averaged over 5 simulation repeats, and the averaged curves are presented with the corresponding error bars for monomers, , dimers, , trimers, , and all kinetic units, . The fitted curves are also presented for monomers, *n*_1,fit_, dimers, *n*_2,fit_, trimers, *n*_3,fit_, and all kinetic units, *n*_c, fit_.

Our model predicts a variety of aggregate structures, from amorphous to fibrillar. In the studied concentration range, we observed only downhill aggregation that leads to clusters with different internal structures determined by the model parameters. The question arises whether the aggregation kinetics correlate with final aggregate structures, in particular, whether different structures appear through different molecular pathways. Visual inspection of the simulation trajectories showed the presence of cluster fragmentation and that small aggregates grow by monomer addition and coallescence. Accordingly, we have developed a three-parameter model that captures the time evolution of the average number of monomers, dimers, trimers, and the total number of clusters including monomers: , and . This model fits the kinetic curves well, but the quality of the fit deteriorates when glass-like and fibrillar structures are formed, see Table 2, Figure 5, and Supporting Information Figure S6. This may be an indication of pathway differences that are not captured by our simple model.

**Table 2:** Example Kinetic Parameters *k*^+^, *k*^–^, and *q*, for Amorphous, Amyloid-Glass, and Fibril Aggregation as Functions of Model Parameters^a^

	σ_SS_/nm	*r*_BS_^0^/nm	*k*^+^/(nm^3^/ns)	*k*^–^/(10^–5^ ns^–1^)	*q*	cost
*amorphous	0.37	0.4	7.84	10.69	3.07	740.5
amorphous	0.45	0.4	8.19	2.22	2.79	921.4
*amyloid-glass	0.43	0.55	9.13	9.36	2.84	1098.2
*amyloid-glass	0.45	0.55	7.65	16.19	3.26	3083.4
fibril	0.37	0.7	6.33	3.49	3.56	1766.1
*fibril	0.45	0.7	9.04	2.02	2.39	1489.1

a The asterisks indicate the fits for the kinetic data illustrated in Figure 5 and Supporting Information Figure S6. There is no apparent correlation of the kinetics parameters with the final aggregate structure. The quality of the fit is quantified by the final value of the cost function (last column). The quality of the fit is best for amorphous structures and decreases when glass-like and fibrillar structures are formed. Note an outlier at the second amyloid-glass entry.

Mansbach and Ferguson^19^ found that the downhill aggregation kinetics of their coarse-grained peptide model were well-described by the Smoluchowski kinetics, where irreversible aggregation occurs with the same rate constant for all elementary steps, *C*_*i*_ + *C*_*j*_ → *C*_*i*+*j*_. Here, we found that a Smoluchowski-type model is not sufficient for downhill aggregation of short peptides, and that cluster fragmentation, C_*i*+*j*_ → C_*i*_ + C_*j*_, needs to be accounted for. Knowles et al.^32^ found pronounced effects of fibril fragmentation on nucleated aggregation. Our work shows that fragmentation needs to be accounted not only for fibrillar but also for amorphous and glass-like structures. Yoshimura et al.^33^ argued that aggregation kinetics may correlate with the final aggregate structure. Here, we did not find a clear indication of such correlation.

Despite much work, there are still many outstanding issues related to peptide aggregation and fibrillation. For instance, the aggregation pathways and aggregate structures are conditioned on the temperature, concentration,^17,34^ pH,^35^ and other external factors.^36^ This work, however, focuses on general internal peptide features that affected fibrillation. The simplest one-bead-per-residue models with isotropic potentials reconstruct aggregation with its kinetic and structural variability^6^ but are not able to reproduce fibril formation.^14^ This provides a clue that fibrillation is conditioned on more sophisticated interactions between peptides.

The previously developed coarse-grained models allow for fibril formation by incorporating directional interactions which account for hydrogen bonding and dipole–dipole interactions. These directional interactions may be treated in several ways; one of them is based on geometrical constraints for interacting superatoms as in the case of the tube model proposed by Hoang.^37^ Auer and co-workers used the tube model to study amorphous to fibril transitions and concluded that, indeed, aggregate reorganization is ruled by the hydrogen bonding defined by orientation-based potentials.^38^

Another way to handle directional peptide interactions is by placing partial charges on backbone superatoms. This method was used by Caflish and co-workers and by Ganesan and Matysiak in the WEPROM model.^12,39^ Both models show a tendency to fibrillation. Ganesan and Matysiak argue that these dipole interactions are a key factor for fibrillation.

A second factor pointed out as crucial for fibrillation is β-sheet propensity, i.e., the tendency of a peptide chain to stay in an elongated, β-strand-like conformation.^4^ Usually, the β-sheet propensity is defined by the backbone dihedral potential. Bellesia and Shea studied the impact of the dihedral constant on fibrillation.^13^ They found that amorphous aggregates are formed for low β-sheet propensity whereas the high values of the dihedral constant, stabilizing the *trans* conformation, promote the formation of ordered oligomers, whose further growth leads directly to the β-barrels or fibrils.

In this landscape, our model provides a novel insight into the fibrillation process, pointing to the presence of side chains being a sufficient factor for fibril formation, despite the absence of directional potential terms or conformational constraints. As we have shown previously, the nondirectional backbone attraction is responsible for aggregation and, together with chain stiffness, determines the aggregate structures from amorphous to crystal-like.^7,14^ In the present work, we demonstrated that the addition of the side chains turns the ordered, cylindrical aggregates into fibrils, mainly promoted by the long side chain–backbone bonds. However, in our model, the fibril-capable systems have an *r*_BS_^0^ value longer than the distance between the Cα carbon and side chain mass center in real peptides which is usually smaller than 0.40 nm.^40^ This discrepancy is justified by the coarse-grained nature of our model. The side chain–backbone distance contributes to the side chain anisotropy, and the excessive value of *r*_BS_^0^ enhances the axial symmetry of the side chain. The issue of the side chain–backbone distance brings out the general question of the intrinsic peptide features enabling fibrillation. Nevertheless, our model shows that the structure of peptide self-assemblies may be modified only by the side chain geometry. These results indicate that the fibrillation process may be quite generic and not restricted to proteins and peptides.

## 4 Conclusions and Outlook

In this work, we have developed a minimal bead-based model that reproduces peptide fibrillation. Notably, this implicit solvent two-bead-per-residue model does not involve directional interactions, which suggests that the side chain geometry is the basic factor inducing fibrillation. Obviously, directional interactions, hydrogen bonds, and dipole–dipole interactions are necessary for sequence-specific peptide models to reproduce more realistic fibrillar features, especially the transition from β-hairpin-like structures to common cross-β pattern of fibrils. Nevertheless, our models show that the residue anistropy is sufficient for fibrillation so that interaction directionality is a modifier to the basic geometrical effect. To the best of our knowledge, it is the first time when the residue geometry was studied in the context of aggregation. Besides its biological relevance, this study contributes to the problem of coarse-grained model design, pointing out to the importance of geometrical parameters for the model behavior.

In our model, aggregate morphology is determined by side chain geometry. The distance between side chain mass center and backbone mass center is a primary factor, whereas the side chain size is a secondary factor. This simple model reproduces the structural variability of peptide aggregates, including amorphous aggregates, amyloid-glass structures, fibrils, and the annular structures reported experimentally. Peptide pairs are the basic motif found in amyloid-glass structures and more ordered aggregates. The peptide arrangement in such pairs is quite similar to that in a β-hairpin, as the peptide chains are connected by backbone interactions whereas the side chains are located outside. Amyloid-glass structures feature relatively high potential energy, but they are stable within our simulation time. Systems with fibrillar propensity, prepared in an initial, amyloid-glass aggregate structure, undergo quick reorganization to fibrils. On the other hand, the system with an amorphous propensity, prepared in an initial amyloid-glass aggregate structure, shows slower losses of the inner parallel peptide arrangement in time. Annular structures, that are similar to amyloid ion channels formed by Aβ oligomers, are stable for the longest backbone–side chain distance and for small side chains. For the larger side chains, initial annular structures collapse into double-ribbon fibrils.

We found that a simple rate equation model with fast monomer association and dissociation kinetics captures the essential aggregation kinetics for all types of aggregates: amorphous, glass-like, and fibrillar. This indicates that the kinetics may not be a good predictor of the final aggregate morphology. We believe that a further search for more sophisticated aggregation models is worthwhile. In particular, a comprehensive model of fragmentation of clusters, from amorphous to fibrillar, is desirable.

## Supporting Information Available

Additional details of the working method and further results on cluster mophology (PDF)
