# Supplementary material for: Side Chain Geometry Determines the Fibrillation Propensity of a Minimal Two-Beads-per-Residue Peptide Model
Source: J Phys Chem B. 2022 Aug 2;126(31):5772–80. doi: 10.1021/acs.jpcb.2c03502 (PMC9376954; doi:10.1021/acs.jpcb.2c03502)
Supplement: Supplementary file 1 — jp2c03502_si_001.pdf [file jp2c03502_si_001.pdf]

Supporting Information

for

Side-Chain Geometry Determines the Fibrillation Propensity of a Minimal Two-Beads-per-Residue Peptide Model

Beata Szała-Mendyk,[†] Andrzej Molski[‡]
Adam Mickiewicz University in Poznań, Faculty of Chemistry,
Uniwersytetu Poznańskiego 8, 61-614 Poznań, Poland
[†]beata.szala@amu.edu.pl, [‡]andrzej.molski@amu.edu.pl

Contents

S1 Additional details of the working method **S2**

S1.1 Cut-off distance for cluster definition S2

S1.2 Local backbone positional order, C_{BB} S2

S2 Further results **S4**

S2.1 Cluster morphologies S4

S2.2 Aggregation kinetics S6

S1 Additional details of the working method

S1.1 Cut-off distance for cluster definition

The definition of a cluster is based on a cut-off distance: a peptide belongs to a cluster if the distance between an atom of this peptide and an atom of a different peptide in the cluster is equal to or less than 5.5 Å. This value is taken from the first maximum in the inter-molecular distances histograms, see Figure S1.

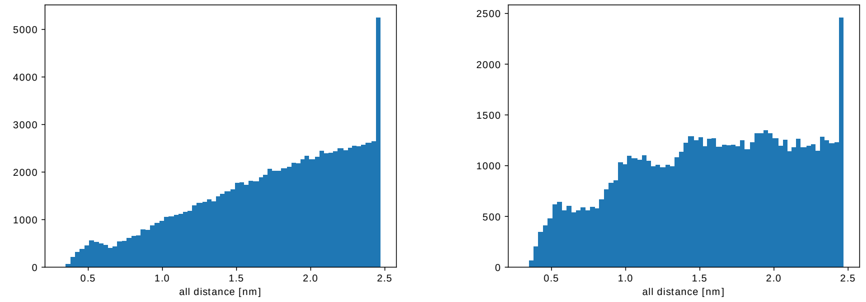

Figure S1: Histograms of the inter-molecular distances between any two super-atoms for maximal, $M = 72$, amorphous aggregate (left panel) and fibril (right panel), respectively. Both systems have the same side chain Lennard-Jones radii, $\sigma_{\mathrm{SS}} = 0.37$. The equilibrium bonds between the side chain and backbone super-atoms are: $r^0_{\mathrm{BS}} = 0.40$ nm (left panel), $r^0_{\mathrm{BS}} = 0.70$ nm (right panel).

S1.2 Local backbone positional order, C_{BB}

The most ordered structures obtained in our simulations show positional order: there are certain distances with large probabilities of finding neighbor peptides. This is clearly seen in the histograms of the inter-molecular distances between any two atoms, Figure S1, and the histograms of distances between backbone super-atoms, Figure S2. Based on those histograms we define shells, as the separation ranges with the largest probability of finding neighbor peptides. The first shell includes the first histogram maximum at $r \simeq 0.55$ nm, which was common for all observed structures: amorphous, glass-like, and fibrillar. The first maximum does not distinguish different structures, hence, we decided to calculate positional order for the peptides within distances corresponding to the second, third and fourth shell in fibrils, i.e. for peptides within $0.8 \le r \le 2.4$ nm.

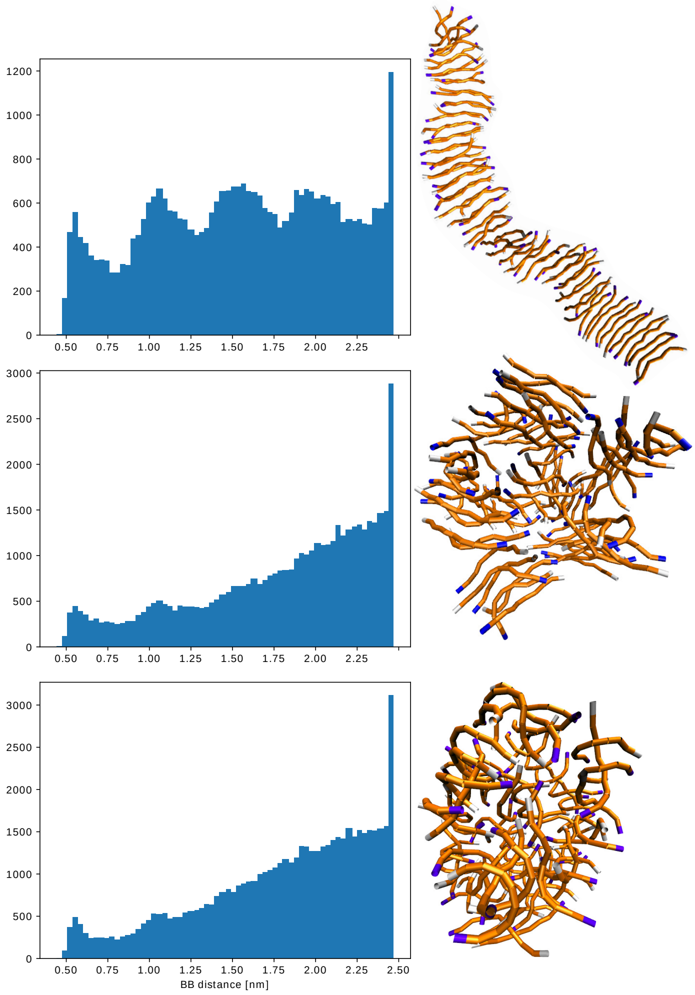

Figure S2: Inter-molecular backbone distance histograms for three different maximal,i.e. $M = 72$, clusters: one-ribbon fibril (top panel), amyloid-glass (middle panel) and amorphous cluster (bottom panel) with the corresponding example structures. All systems have the same side chain Lennard-Jones radius, $\sigma_{\text{SS}} = 0.43$ nm. The equilibrium bonds between side chain and backbone super-atoms are: $r^0_{\text{BS}} = 0.70$ nm (top panel), $r^0_{\text{BS}} = 0.55$ nm (middle panel), $r^0_{\text{BS}} = 0.40$ nm (bottom panel)

S2 Further results

S2.1 Cluster morphologies

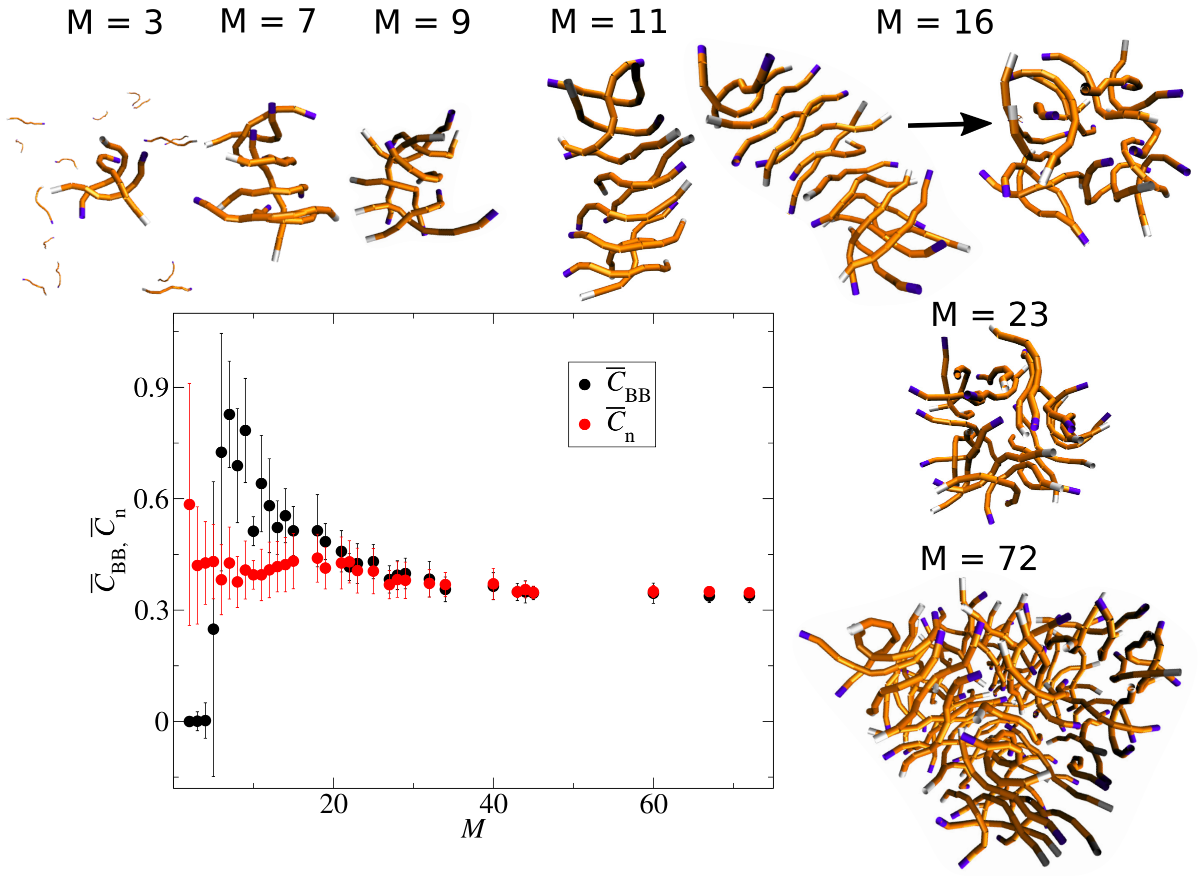

Figure S3: Average end-to-end correlation parameter, $\overline{C}_{\mathrm{n}}$, and backbone correlation parameter, $\overline{C}_{\mathrm{BB}}$, as a function of the aggregate size, M, for the side chain super-atom LJ radii $\sigma_{\mathrm{SS}} = 0.37$ nm and the length of the backbone - side chain super-atoms bond $r^0_{\mathrm{BS}} = 0.400$ nm.

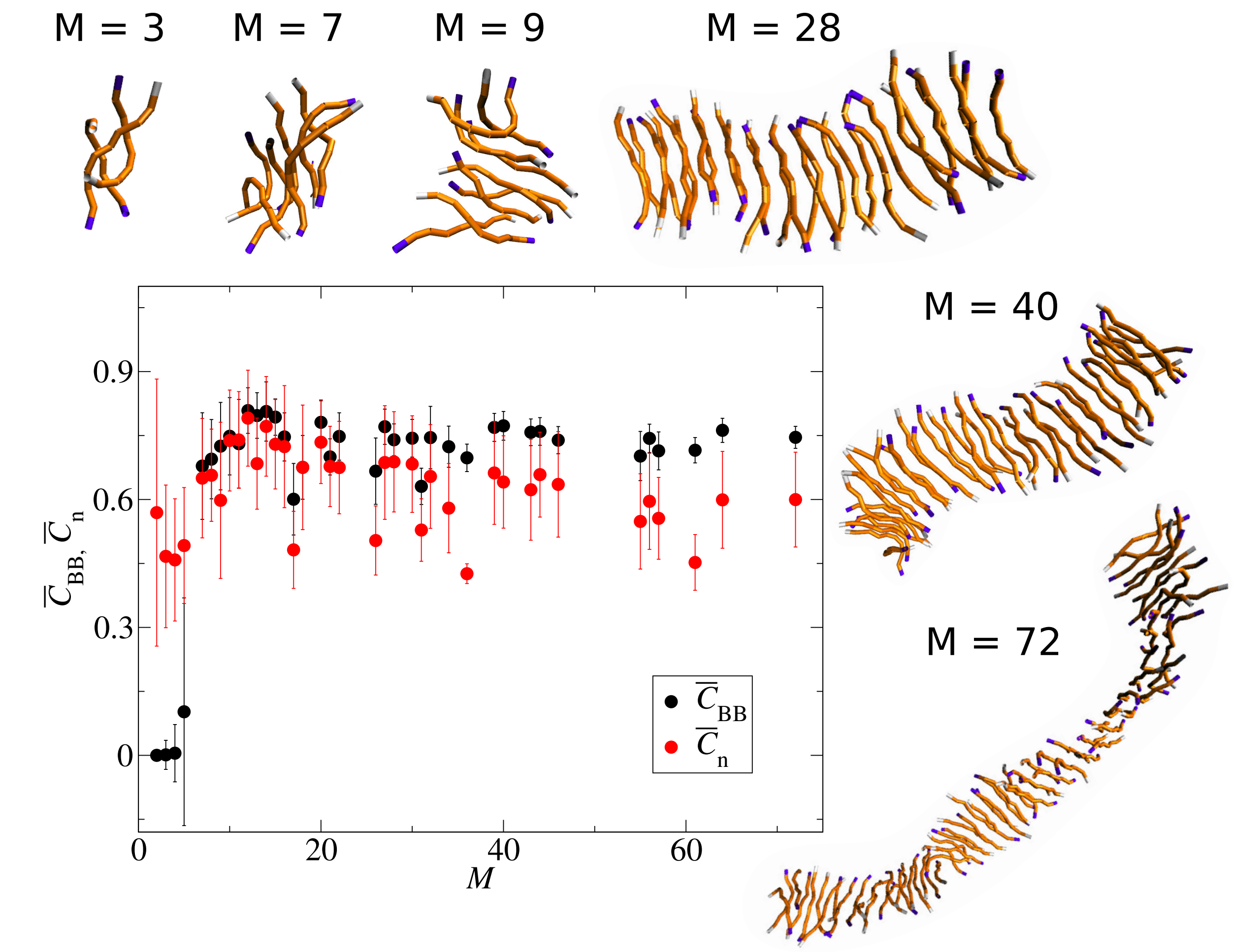

Figure S4: Average end-to-end correlation parameter, $\overline{C}_{\mathrm{n}}$, and backbone correlation parameter, $\overline{C}_{\mathrm{BB}}$, as a function of the aggregate size, M, for the side chain super-atom LJ radii $\sigma_{\mathrm{SS}} = 0.37$ nm and the length of the backbone - side chain super-atoms bond $r^0_{\mathrm{BS}} = 0.400$ nm.

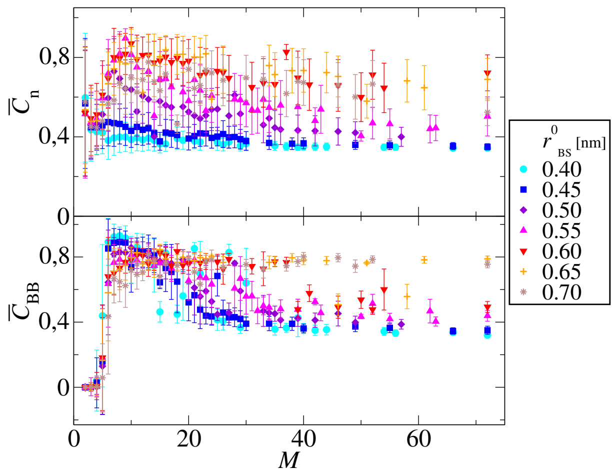

Figure S5: Average end-to-end correlation parameter, $\overline{C}_{\mathrm{n}}$, top panel, and backbone correlation parameter, $\overline{C}_{\mathrm{BB}}$, bottom panel, as a function of the aggregate size, M, for side chain super-atom LJ radius $\sigma_{\mathrm{SS}} = 0.41$ nm and different length of the backbone - side chain super-atoms bond as indicated. Error bars correspond to the standard deviation of the sample.

S2.2 Aggregation kinetics

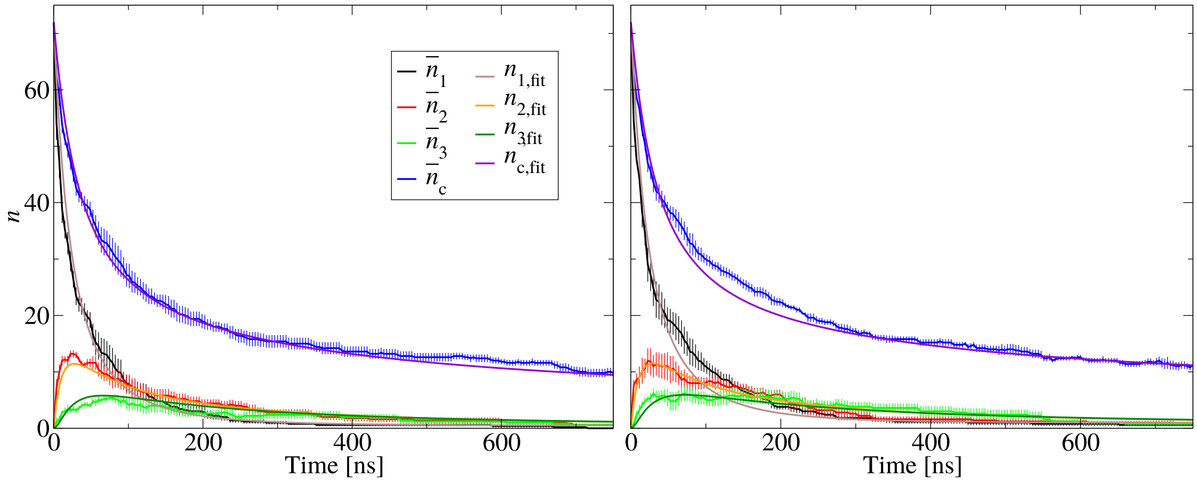

Figure S6: Kinetic plots for two amyloid-glass systems, left panel: $\sigma_{SS} = 0.43$, $r^0_{BS} = 0.55$ nm, right panel: $\sigma_{SS} = 0.45$, $r^0_{BS} = 0.55$ nm. Data from the MD simulations are averaged over 5 simulation repeats, and the averaged curve is presented with the corresponding error bars for monomers, $\overline{n}_1$, dimmers, $\overline{n}_2$, trimers, $\overline{n}_3$, and all kinetic units, $\overline{n}_c$. The fitted curve are also presented for monomers, $n_{1,\text{fit}}$, dimmers, $n_{2,\text{fit}}$, trimers, $n_{3,\text{fit}}$, and all kinetic units, $n_{c,\text{fit}}$.
